# Application of Artificial Neural Network Models in Segmentation and Classification of Nodules in Breast Ultrasound Digital Images

**DOI:** 10.1155/2016/7987212

**Published:** 2016-06-16

**Authors:** Karem D. Marcomini, Antonio A. O. Carneiro, Homero Schiabel

**Affiliations:** ^1^Department of Electrical and Computer Engineering, University of São Paulo, 400 Avenida Trabalhador São-Carlense, 13566-590 São Carlos, SP, Brazil; ^2^Department of Physics, University of São Paulo, 3900 Avenida Bandeirantes, 14040-901 Ribeirão Preto, SP, Brazil

## Abstract

This research presents a methodology for the automatic detection and characterization of breast sonographic findings. We performed the tests in ultrasound images obtained from breast phantoms made of tissue mimicking material. When the results were considerable, we applied the same techniques to clinical examinations. The process was started employing preprocessing (Wiener filter, equalization, and median filter) to minimize noise. Then, five segmentation techniques were investigated to determine the most concise representation of the lesion contour, enabling us to consider the neural network SOM as the most relevant. After the delimitation of the object, the most expressive features were defined to the morphological description of the finding, generating the input data to the neural Multilayer Perceptron (MLP) classifier. The accuracy achieved during training with simulated images was 94.2%, producing an AUC of 0.92. To evaluating the data generalization, the classification was performed with a group of unknown images to the system, both to simulators and to clinical trials, resulting in an accuracy of 90% and 81%, respectively. The proposed classifier proved to be an important tool for the diagnosis in breast ultrasound.

## 1. Introduction

According to the American Cancer Society, more than 178,000 women are affected by breast cancer every year; international statistics reports estimated 1,152,161 new cases annually. This form of the disease is the leading killer of women between 40 and 55 years old and is the second leading cause of death overall in women [1]. Due to this, screening techniques allowing early detection and diagnosis have been studied in order to increase the chances of survival using less aggressive treatment [2, 3].

Among the screening techniques currently available, mammography is the most often used, considered as the gold standard to breast tumor detection. However, this procedure is less effective when investigating dense breasts due to relatively high false negative rates [1]. Moreover, the number of unnecessary biopsies is very large and can lead to changes in the parenchyma making it difficult to read subsequent mammographic images [2].

In recent years, ultrasonography has proven a valuable technique used as an adjunct to conventional mammography for the detection and classification of breast lesions [1]. This procedure has been used to obtain additional diagnostic information, in order to reduce the number of unnecessary biopsies and assist with more accuracy the diagnosis of simple cysts (around 96–100% of efficacy when both of these techniques are used together) [4]. An additional advantage of ultrasound is that it does not use ionizing radiation and therefore is useful especially for younger patients who tend to have dense breasts [5].

The detection of abnormalities in medical images is a procedure prone to errors, even for qualified radiologists, due to the subjectivity in defining boundaries, overlap between benign and malignant characteristics, and the presence of artifacts that may confuse the diagnosis [6]. In order to increase diagnostic accuracy and minimize such errors, computational tools have been developed to provide a second opinion for the specialist and assist in early detection of breast cancer. In this context, this work aims to develop a tool to aid the diagnosis based on the automatic detection of lesions in ultrasound images and the consequent classification of such finding as clinically suspicious (malignant) or not (benign) considering an analysis of their morphological characteristics.

## 2. Materials and Methods

The database used in this research consisted of two distinct sets of breast ultrasound images. The first corresponds to images from breast phantoms and the second from conventional clinical examinations.

### 2.1. Database

**Figure 1:**
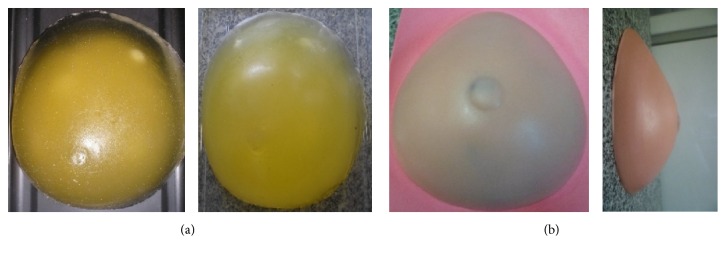
Breast biopsy phantoms designed for ultrasound images: (a) BB-1 model from ATS Laboratories and (b) models developed by Vieira et al. [7].

The phantom images were acquired from tests performed by the Group of Innovation in Medical Instrumentation and Ultrasound (GIIMUS) from University of São Paulo, Brazil. The phantoms used in this research were BB-1 model (breast biopsy phantom, ATS Laboratories) and models previously developed by Vieira et al. [7]. All the phantoms were made of an acoustically tissue mimicking material and have a shape similar to the breast of an adult woman.  Figure 1 illustrates few examples of these phantoms.

**Figure 2:**
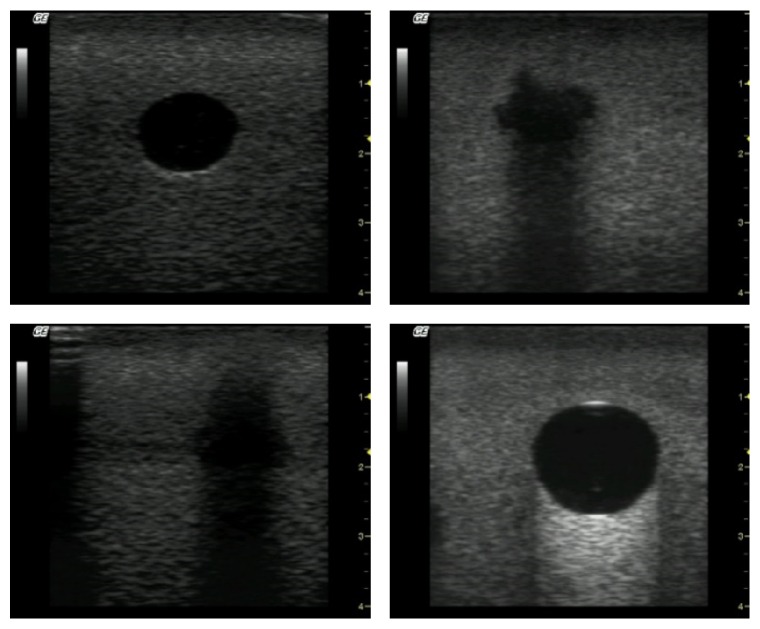
Ultrasound images obtained from exposures of the phantoms shown in Figure 1.

All these phantoms were submitted to ultrasound beams from a GE Logic-Book XP portable device, operating in the frequency range of 1–10 MHz. A total of 144 phantom images in B-mode were acquired as those illustrated in Figure 2.

**Figure 3:**
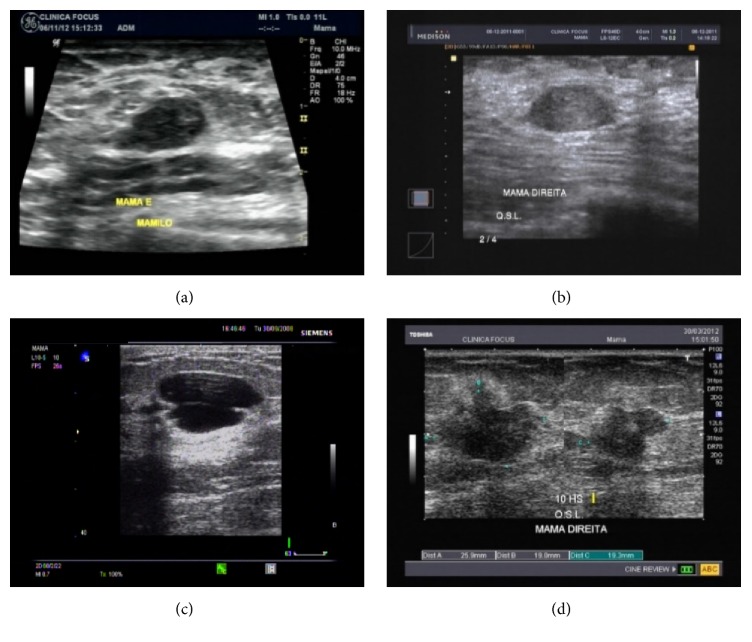
Breast clinical B-mode image acquired from (a) General Electric Logiq P5, (b) Medison X8, (c) Siemens G50, and (d) Toshiba Nemio 30.

Two medical centers of imaging diagnosis at São Carlos, SP, Brazil, provided clinical ultrasound images. Four devices were used in the process of obtaining these images, Siemens G50, Medison X8, Toshiba Nemio 30, and General Electric Logiq P5, considering a broadband linear transducer of 7.5–10 MHz frequency range. A total of 123 images were acquired during imaging routine procedures.  Figure 3 shows some examples of B-mode ultrasound images acquired with such different equipment.

## 3. Images Processing

For each image, an experienced radiologist performed the analysis in order to detect lesions with suspicious appearance and then selected the regions of interest (ROIs). These ROIs had rectangular shape and included the lesion and surrounding tissue. Therefore, such a procedure resulted in 173 ROIs corresponding to actual clinical ultrasound images and 144 from phantoms images.

In order to remove noise and to smooth the image components, ROIs have been preprocessed by a Wiener filter, followed by the contrast enhancement (image equalization) and median filter.

Based on the variability in segmentation techniques, the efficacy was evaluated taking into account some techniques applied in order to highlight the lesion: active contour [14], region growing [15], fuzzy *c*-means [16], *k*-means [17], and SOM neural network [18, 19].

A postprocessing technique was then applied aiming to improve the segmentation quality since many pixels were verified to be disconnected from the actual lesion after the use of some of the techniques mentioned above. As a consequence, this effect has produced a more spiculated and noisier appearance than the nodule actually had. In addition, internal valleys were identified. Therefore, artifacts disconnected from the object of interest were eliminated and the internal valleys have joined the region [20].

## 4. Features Extraction and Selection

Feature extraction in digital images is a critical step for identifying objects. In most cases, the use of more than one measure is required in order to decide to which class the pattern belongs. The most common is to extract from each sample several measures and then represent them through a vector, which will serve as an input to the classifier [21].

In general, benign tumors correspond to softer shapes and malignant tumors tend to have irregular edges [10]. Thus, based on the shape of the lesion as previously reported [22], 24 morphological features were extracted from each one.

**Figure 4:**
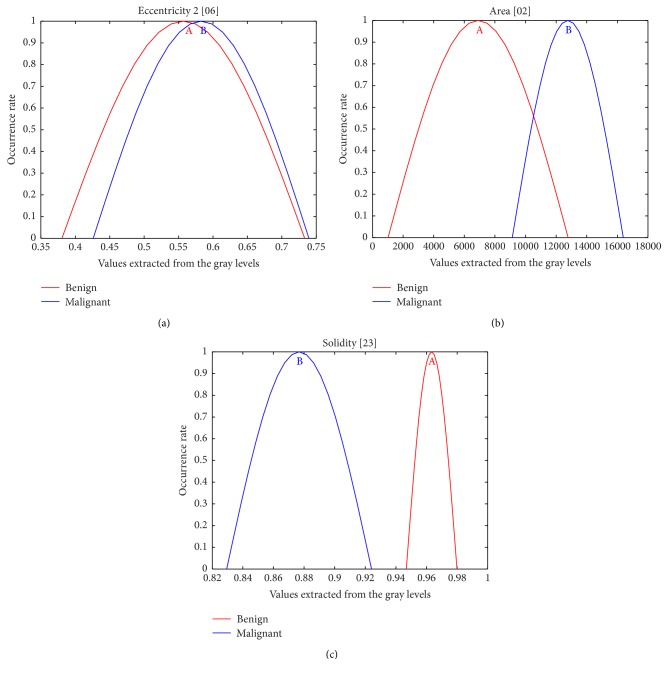
Gaussian distribution curves: (a) almost full overlapping, (b) partial overlapping, and (c) without overlapping between the classes.

However, due to the large number of features considered, Gaussian distribution curves [21] were used regarding this features set optimization, so that it could accurately describe the identified object. In this procedure, each descriptor is normalized and Gaussian curves are generated based on the distribution of values presented for each class (or just two for this work's purposes). The analysis of these curves is performed by visual inspection of some details. The most important is the level of curves intersection: the smaller its occurrence, the higher the probability of such feature representing such a category. Furthermore, the distribution range of the values in the abscissa axis must be checked in order to determine the optimum distance between the classes.  Figure 4 shows some examples of these curves.

## 5. Classification

Multilayer Perceptron (MLP) neural network is a tool frequently used in differentiating between benign and malignant lesions. Its topology consists of sensory units which include the input layer, one or more hidden layers (also known as intermediary), and an output layer [23]. The learning process is supervised; that is, the desired outputs are required. A supervised learning algorithm analyzes, through comparative actions between inputs and the desired output, the training data and produces an inferred function, which can be used for mapping new examples. An optimal scenario will allow for the algorithm to correctly determine the class labels for unknown instances.

After performing comparisons in the learning method (the backpropagation), the synaptic weights are adjusted continuously reaching for convergence. In this step, the discrepancy between the responses produced by the network and the desired signal is evaluated. The network adjusts the values of the synaptic weights and this process is finished only when the error assumes an acceptable value [23].

The cross-validation method is used to evaluate data generalization. This procedure was performed through random partitioning of the dataset into two subsets: training and test [23]. The training was accomplished only in phantom images due to the low number of malignant cases in actual clinical exams. Thus, 144 ROIs from phantoms images were used for classification, 72 corresponding to benign and 72 to malignant images. From these 144 images, 70% were designated for training and 30% for validation.

## 6. Results and Discussion

**Figure 5:**
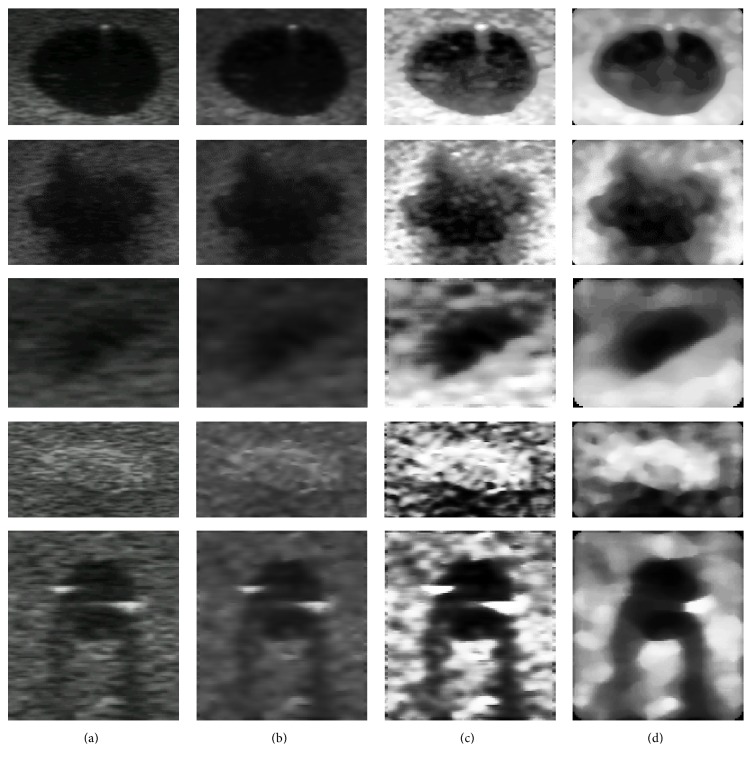
ROIs from breast phantom images: (a) original image; (b) after Wiener filtering; (c) with equalization; and (d) after being submitted to a median filter.

**Figure 6:**
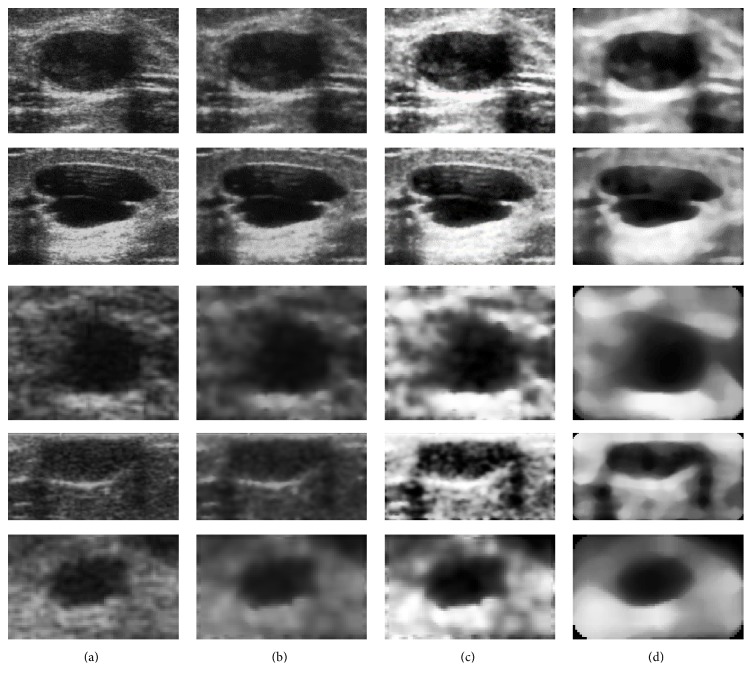
Preprocessing application to ROIs from actual breast ultrasound images: (a) original image; (b) after Wiener filtering; (c) with equalization; and (d) after being submitted to a median filter.

The first computational test was performed on a set of 80 ROIs from phantom images and 50 from actual clinic ones. After the ROI selection, the following preprocessing techniques were applied: Wiener filter, equalization, and median filter. Figures 5 and 6 show some examples of this preprocessing effect on phantom and clinical images.

**Figure 7:**
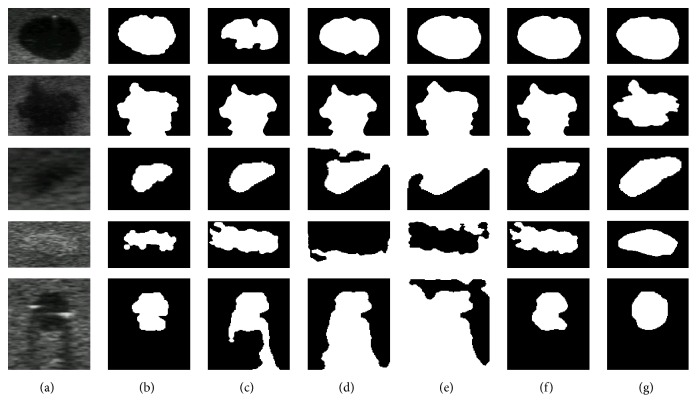
Results from tests with phantom images: (a) original ROI; segmentations by (b) active contour, (c) region growing, (d) fuzzy *c*-means, (e) *k*-means, (f) SOM, and (g) the radiologist delimitation.

In the second step, some segmentation techniques (as those previously mentioned in Section 3) were applied in order to precisely delineate the lesions with smooth and regular edges. The active contour was the only technique that did not use the preprocessing. However, it was necessary to apply opening and closing morphological operators after segmentation to smooth the segmented lesion edge [22]. Manual segmentation provided by an experienced radiologist as well as some examples of the effects of segmentation techniques application and postprocessing on phantom images can be seen in Figure 7.

**Figure 8:**
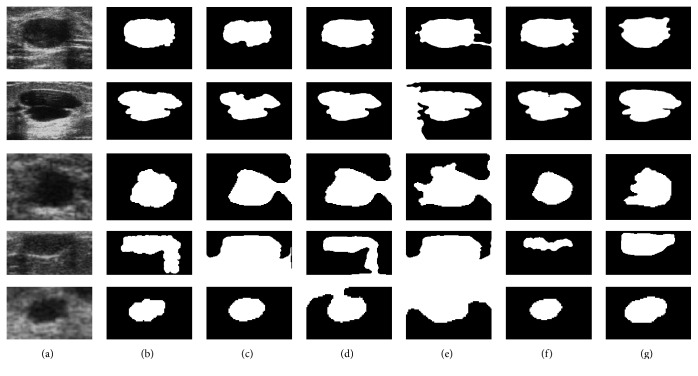
Results from tests with actual images: (a) original ROI; segmentations by (b) active contour, (c) region growing, (d) fuzzy *c*-means, (e) *k*-means, (f) SOM, and (g) delineation by the radiologist.

In order to confirm the validation of these procedures also to actual clinical images, those same techniques were applied and similarly evaluated. Some corresponding results are shown in Figure 8.

Due to the visual subjectivity in evaluation to find the most accurate detector, measures were determined to quantify the distance between the edge automatically defined and that manually delineated by an experienced radiologist. Hence, ten measures were evaluated, according to the descriptions in [24–26]. The calculated values for the phantoms images dataset are shown in Table 1 while Table 2 reports those values but they were calculated for the actual breast images dataset.

Based on these data, the advantage of segmentation by active contours and by the SOM neural network can be noted in comparison to the others. In addition, when comparing both methods with the delimitation by the radiologist, they reported greater accuracy and low error rates. Thus, both detectors were tested individually to gather the best classification results with phantoms as well as actual clinical images.

The first step was the extraction of 24 morphological features [22] only for the 144 phantoms images (72 corresponding to benign signals and 72 to malignant ones). Then, the most relevant features were selected by means of Gaussian distribution curves. Among the 24 curves produced, those not evidencing appropriate visual results, that is, those with fully or partially overlapping areas, were discarded. Just 8 Gaussian distributions provided good partition for both the active contour segmentation and SOM, as shown in Table 3.

Each of the 8 selected features by the Gaussian distribution curves was individually introduced to MLP, but the results achieved were not significant. Thus, tests were performed with all possible combinations.

The network topological configuration was continuously adjusted during the training process. The amount of neurons in the single hidden layer varied from 1 to 9, and the learning rate was constantly adjusted between 0.1 and 0.9. During this step, 70% of the data were allocated to training and 30% to the validation procedure.

The topology that achieved the best result for each detector is described in Table 4. The accuracy rate in classification when the lesion was segmented by SOM was 94.2% and it was 95.6% when the lesion was segmented by active contour.

**Figure 9:**
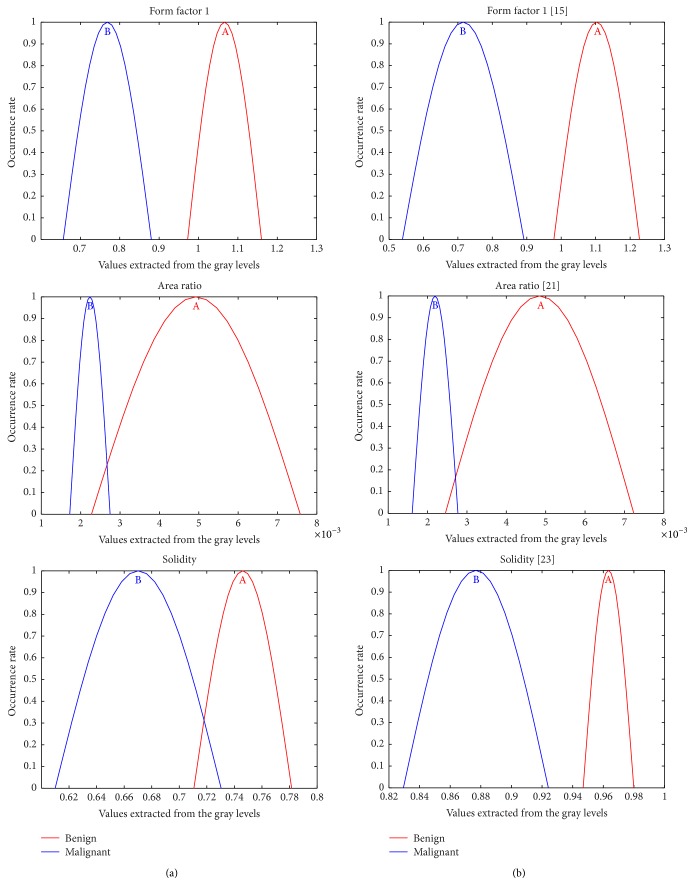
The best results for extracted descriptors from Gaussian curves distribution. (a) Segmentation by active contour; (b) detection by the SOM network.

Figure 9 illustrates the respective Gaussian distribution curves used for determining each descriptor selected by their overlapping analysis.

After completing the classification procedure, we obtained the values of true positive (TP), false positive (FP), false negative (FN), and true negative (TN), as shown in Table 5.

**Figure 10:**
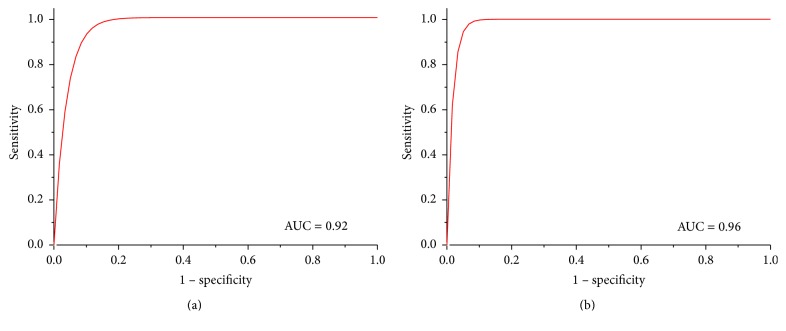
ROC curves obtained from the classification using MLP with US phantoms images. Segmentation by (a) SOM network and (b) active contour.

In Figure 10, ROC curves regarding the classification of phantom images with MLP are shown as for segmentation by SOM network (a) as well as by active contour (b). The values calculated for the areas under the curve (AUC) are displayed in their respective graphics.

The performance of this classifier was compared to others previously described by correlate literature with similar purposes.  Table 6 shows the data previously presented by each one of those studies in comparison to our results mainly in terms of accuracy, sensitivity, and specificity rates.

Both classification proposals, with the contour segmented by SOM (case 1) and active contour (case 2), have yielded high sensitivity and specificity in breast lesions classification, similar to the works considered in Table 6. The same accuracy was achieved for both cases and it was higher than all the results presented by such works. In our study, the sensitivity was higher in case 1, while the specificity was higher in case 2.

Additionally, it is important to stress that in our study the training and validation procedures were performed with phantom images, due to the small set of clinical images corresponding to the class “malignant.” These phantom images had structures of interest with a relatively regular shape and in some cases they were easily segmented. This aided in achieving high accuracy in both cases (for benign and for malignant simulated structures). In order to evaluate whether the classifier is able to generalize and reach similar results when applied to actual clinical images, tests with the second dataset were performed, taking into account the fact that it was trained with phantom images which have slightly different intrinsic characteristics. With this purpose, an experienced radiologist determined 173 ROIs which were automatically segmented by both techniques (i.e., active contour and SOM). Then, measures of solidity, area ratio, and form factor were extracted from each ROI. Finally, these data were used by the neural network classifier with the topological configuration providing the best result for each detector (shown in Table 4). The results obtained in this classification are given in Table 7.

**Figure 11:**
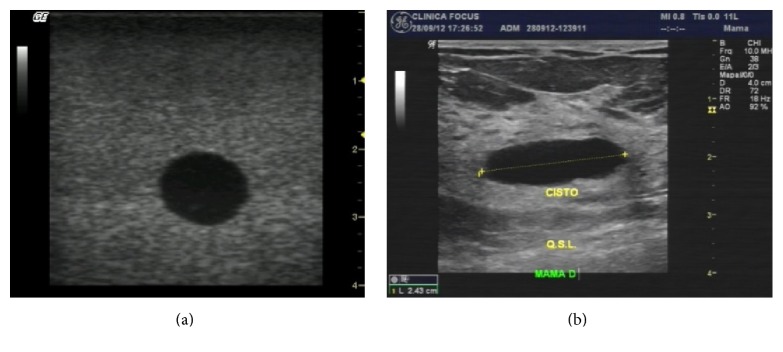
Example of a morphological difference between (a) phantom image and (b) actual clinical exam.

The high FP rates are largely related to morphological differences between phantom images and actual ones, mainly the simulated structures in the phantoms under test with more rounded shapes. As a consequence, clinical images with elongated shape tumors were erroneously classified.  Figure 11 illustrates an example of such feature.

**Figure 12:**
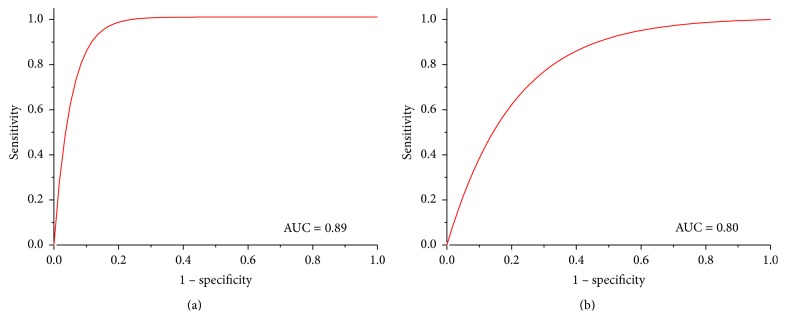
ROC curves obtained from the classification with actual clinical images. (a) Segmentation by SOM network; (b) segmentation by active contours.

Even with these morphological differences between the types of images, the classifier achieved good data generalization, reaching 100% of sensitivity and 78% of specificity when using segmentation by SOM. In the classification after segmentation by active contour, the sensitivity rate decreased significantly (only 63%) though specificity was almost the same: 79%. This difference is evident when the area under the curve is calculated; corresponding results are shown in Figure 12.

## 7. Conclusions

The overlap of benign and malignant characteristics in interpreting ultrasound images turns the process subjective and tends to complicate the diagnosis of breast lesions. For this reason, CAD schemes have emerged to improve the analysis of the radiologist by means of computerized characterization.

Some flaws however often arise in many CAD schemes when evaluating images from breast ultrasound acquisitions mainly due to speckle noise influence on the lesions boundaries definition. This is the reason of applying preprocessing techniques before the segmentation procedure.

Testing five detectors techniques and measuring how close they were relative to the ground truth, only the SOM network and the active contour yielded significant accuracy rates.

A differential of active contour technique is not requiring the preprocessing and the use of morphological operators to smooth edges. Nevertheless, this final smoothing caused some changes in lesion limits, influencing the classification step. Moreover, its algorithmic complexity requires many numerical operations and iterations until convergence of data has been reached. Consequently, this leads to a high computational cost, making the processing relatively slow: about 30 seconds for each ROI.

The segmentation by SOM network on the other hand produced smoother contours and faster outcome results allowing a better understanding of the morphological differences between benign and malignant lesions.

Based on such a result, the classification by MLP was performed for both detectors. After extensive tests and topological changes, the classification taking breast phantom images with detection by active contour was more than 95% accurate, a rate higher than that for detection by SOM network (94.24%). However, the index of the classification with detection by active contour decreased significantly when applied to actual clinical images, which has registered an accuracy of 77.5%. In contrast, the classification accuracy rate with detection by SOM was almost 81%. Therefore, the detection using the neural SOM network allowed better accuracy and data generalization when new images were introduced to the classifier.

Another important feature to be stressed is the contribution that breast phantoms ultrasound images have made to this investigation, since they constituted a manageable dataset with known materials and morphological characteristics. Furthermore, as large variations related to the simulated lesion morphology can be provided, the dataset becomes useful for the network training before the tests with actual US images, especially with the new guidelines and recommendations for clinical use of ultrasound elastography.

The system described here has an intuitive and easy interface, providing fast and accurate responses to the specialist. The tool is fully automated but allows, if necessary, user intervention to improve the segmentation process, due to manual change in the maximum and minimum parameters in the training. It is considered that the system has produced good results, acting as an important tool for the aid in diagnosis in breast ultrasound.

## Figures and Tables

**Table 1:** Evaluation metrics applied to phantoms images regarding the five segmentation techniques applied in the tests.

	AOM (%)	AUM (%)	AVM (%)	CM (%)	CP (%)	CR (%)	*Q* (%)	*A* (%)	Err. (%)	FPR (%)
Active contour	83.07	15.44	1.90	88.57	84.55	98.10	83.07	94.73	5.27	0.87
Region growing	75.02	22.50	3.23	83.09	77.49	96.76	75.02	91.51	8.48	1.06
Fuzzy *c*-means	72.38	19.75	18.51	78.03	80.24	81.48	72.38	87.81	12.18	8.02
*k* -means	60.73	9.72	36.78	71.41	90.27	63.21	60.73	77.63	22.36	27.69
SOM	82.50	13.17	5.17	88.05	86.82	94.82	82.50	94.32	5.67	2.02

Required value	100	0	0	100	100	100	100	100	0	0

**Table 2:** Evaluation metrics applied in actual breast images regarding the five segmentation techniques applied in the tests.

	AOM (%)	AUM (%)	AVM (%)	CM (%)	CP (%)	CR (%)	*Q* (%)	*A* (%)	Err. (%)	FPR (%)
Active contour	81.69	8.57	11.53	87.20	91.43	88.47	81.69	95.83	4.17	3.05
Region growing	70.85	19.42	25.55	93.22	67.13	92.07	62.32	89.79	10.21	2.19
Fuzzy *c*-means	59.86	24.51	33.63	71.96	77.41	72.84	57.36	85.19	14.81	10.87
*k*-means	38.92	30.74	57.02	42.26	87.65	45.65	41.61	67.29	32.71	37.89
SOM	75.25	19.75	24.09	93.97	67.46	96.77	65.15	91.33	8.66	0.62

Required value	100	0	0	100	100	100	100	100	0	0

**Table 3:** The most relevant descriptors selected by Gaussian distribution curves for each segmentation method.

Most relevant features	
Active contour	SOM
Perimeter [8]	Perimeter [8]
Compactness [9]	Compactness [9]
Circularity [8]	Circularity [8]
Convexity [9]	Convexity [9]
Form factor [10]	Form factor [10]
Area ratio [11]	Area ratio [11]
Residue [11]	Rectangularity [9]
Solidity [9]	Solidity [9]

**Table 4:** Topological configuration of MLP.

MLP	Active contour	SOM
Inputs	3 (form factor, area ratio, and solidity)	3 (form factor, area ratio, and solidity)
Number of intermediate layers	1	1
Number of neurons in the hidden layer	3	2
Learning rate	0.5	0.5
Number of outputs	2	2

**Table 5:** Evaluation metrics applied to ultrasound breast phantoms images.

Detector	Classes		MLP			
			Accuracy by class			
	Benign	Malignant	TP	FN	FP	TN
Active contour	72	72	70	2	9	63
SOM			68	4	7	65

**Table 6:** Performance of other studies.

	Our study		Yap M. H. and Yap C. H. [12]	Liao et al. [13]	Chang et al. [10]	Alvarenga et al. [11]
	Segmentation using SOM	Segmentation using active contour				
Accuracy	0.924	0.924	0.833	0.866	0.910	0.888
Sensitivity	0.944	0.972	0.780	0.754	0.889	0.860
Specificity	0.903	0.857	0.871	0.951	0.925	0.942
Area under ROC curve	0.920	0.960	0.884	—	0.947	0.920

**Table 7:** Classification of actual clinical images.

Detector	Classes		MLP			
			Accuracy by class			
	Benign	Malignant	TP	FN	FP	TN
Active contour	150	23	15	8	31	119
SOM			23	0	33	117
